# Commissioning measurements on an Elekta Unity MR-Linac

**DOI:** 10.1007/s13246-022-01113-7

**Published:** 2022-03-02

**Authors:** Marcus Powers, John Baines, Robert Crane, Chantelle Fisher, Stephen Gibson, Linda Marsh, Bronwyn Oar, Ariadne Shoobridge, Emily Simpson-Page, Marchant Van der Walt, Glenn de Vine

**Affiliations:** 1 Townsville Cancer Centre, Townsville Hospital and Health Service, Townsville, QLD Australia; 2 College of Science and Engineering, James Cook University, Townsville, QLD Australia; 3 Cancer Care Services, Royal Brisbane and Women’s Hospital, Herston, QLD Australia

**Keywords:** MR-Linac, Commissioning, Elekta Unity

## Abstract

Magnetic resonance-guided radiotherapy technology is relatively new and commissioning publications, quality assurance (QA) protocols and commercial products are limited. This work provides guidance for implementation measurements that may be performed on the Elekta Unity MR-Linac (Elekta, Stockholm, Sweden). Adaptations of vendor supplied phantoms facilitated determination of gantry angle accuracy and linac isocentre, whereas in-house developed phantoms were used for end-to-end testing and anterior coil attenuation measurements. Third-party devices were used for measuring beam quality, reference dosimetry and during treatment plan commissioning; however, due to several challenges, variations on standard techniques were required. Gantry angle accuracy was within 0.1°, confirmed with pixel intensity profiles, and MV isocentre diameter was < 0.5 mm. Anterior coil attenuation was approximately 0.6%. Beam quality as determined by TPR_20,10_ was 0.705 ± 0.001, in agreement with treatment planning system (TPS) calculations, and gamma comparison against the TPS for a 22.0 × 22.0 cm^2^ field was above 95.0% (2.0%, 2.0 mm). Machine output was 1.000 ± 0.002 Gy per 100 MU, depth 5.0 cm. During treatment plan commissioning, sub-standard results indicated issues with machine behaviour. Once rectified, gamma comparisons were above 95.0% (2.0%, 2.0 mm). Centres which may not have access to specialized equipment can use in-house developed phantoms, or adapt those supplied by the vendor, to perform commissioning work and confirm operation of the MRL within published tolerances. The plan QA techniques used in this work can highlight issues with machine behaviour when appropriate gamma criteria are set.

## Introduction

The integration of magnetic resonance (MR) imaging and mega-voltage (MV) beam generation achieved with the Elekta Unity MR-Linac (MRL) (Elekta, Stockholm, Sweden) design provides a leap forward in image-guided radiotherapy. However, with this comes a new set of quality assurance (QA) challenges. Following the on-site construction of the system, Elekta personnel perform device acceptance tests (DAT) that replace conventional linac acceptance tests. Commissioning and beam modelling validation measurements are also performed by Elekta personnel, with Philips staff responsible for MR image quality testing. Following this, a period of internal commissioning and quality assurance occurs during which in-house physicists perform baseline and validation measurements across both MR and MV modalities. Elekta tests incorporate specialised QA devices and analysis software that are not necessarily commercially available. Furthermore, due to the presence of the magnetic field, conventional equipment available to the clinic may not be compatible with the Unity system.

**Fig. 1:**
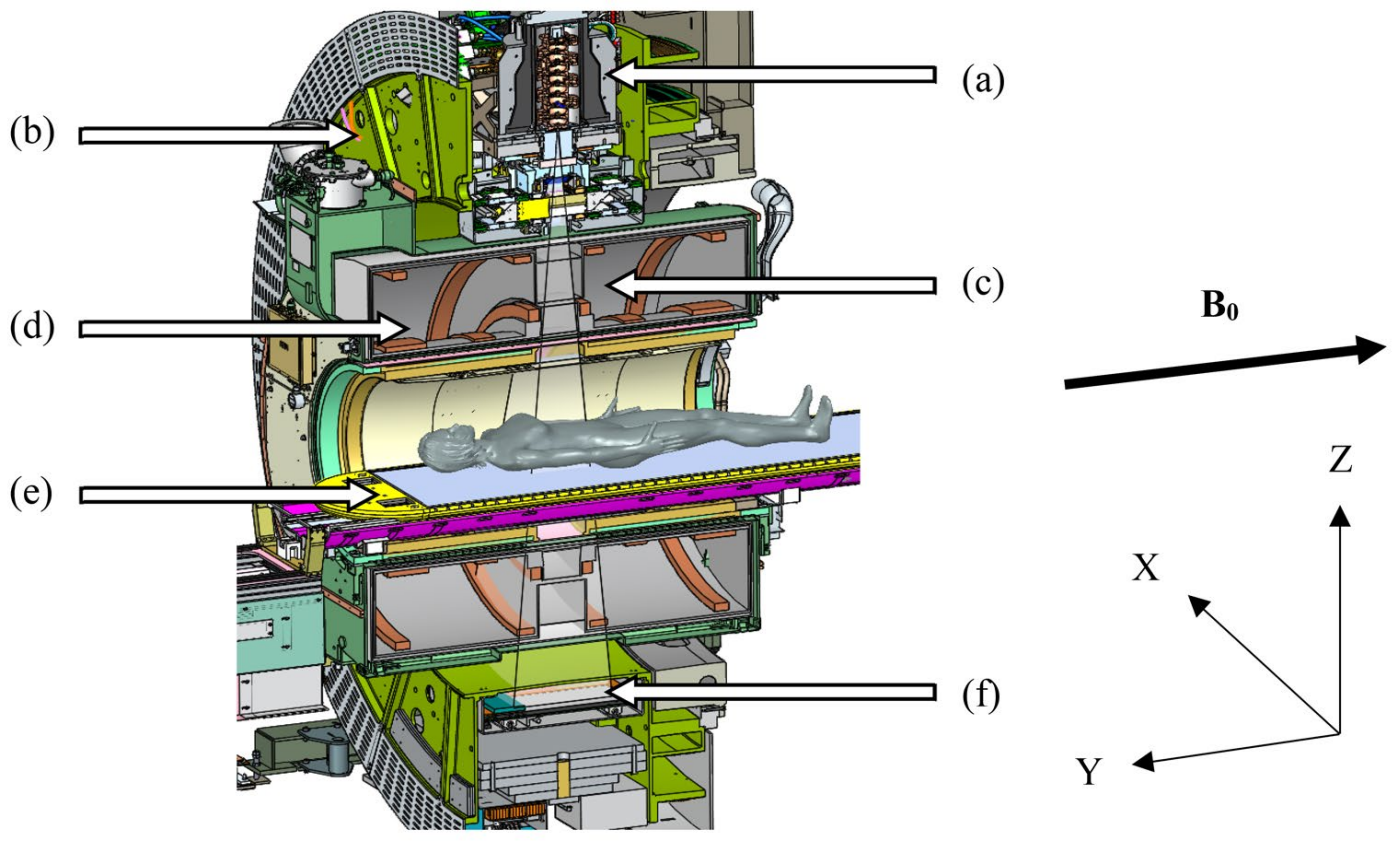
A schematic of the Elekta Unity MR-Linac, courtesy of Elekta, showing (**a**) the straight-through waveguide, (**b**) the gantry ring, (**c**) the primary radiation beam passing through (**d**) the coil system embedded in the magnet cryostat, (**e**) the patient positioning system and (**f**) the MVI. The IEC61217 coordinate system is shown, and for the head-first-supine patient orientation, B_0_ is in the craniocaudal direction (negative Y)

The design of the Unity system has been comprehensively discussed by other investigators [1–5], and the reader is referred to these works for further information. In short, the Elekta Unity MRL is a combination of a modified Philips Ingenia 1.5 T MRI, with a split-coil superconducting magnet and a straight-through linear accelerator. The beam generation system, producing a single 7 MV FFF x-ray source, is mounted on an annular gantry that is free to rotate around a cylindrical cryostat containing the static-field MR coils. Gantry rotation axis and the central axis of the coils are coincident, with the static magnetic field (B_0_) in the negative Y direction (IEC61217) as shown in Fig. 1. For all gantry angles, the beam passes through the cryostat and is perpendicular to B_0_. The angular dependent beam transmission through the cryostat (aluminium annular structure containing liquid helium) is referred to as the cryostat characterisation and will vary between Unity systems mostly due to differences in construction of the cryostat annulus; however, a small component will be from differences in helium fill [3]. A modified Elekta Agility® beam limiting device (BLD) shapes fields ranging from 0.8 × 0.5 to 57.4 × 22.0 cm^2^ at isocentre. At the time this work was conducted the dose-rate at this point was 425.0 MU/min; however, recent upgrades have enabled continuously varying dose rate. The patient positioning system (PPS) is capable of longitudinal movement only and the isocentre is 14.0 cm above the PPS, 143.5 cm from the source. A fixed EPID panel, now called the mega-voltage imager (MVI) [5], diametrically opposite the x-ray source, is capable of MV portal imaging a maximum field size of 22.0 × 9.5 cm^2^, for QA purposes only. A schematic of the MR-linac is shown in Fig. 1, courtesy of Elekta.

Acceptance, commissioning and continuous QA tests, tolerances and frequencies have been described by other authors [3–6]. However, as the Unity has only recently been clinically introduced, the scope of such work is limited. Unity users may encounter challenges performing independent, routine QA tests without further guidance. This may be for a variety of reasons, but in part due to vendor use of specialized equipment and software which may not be readily available to a clinic. Additionally, because of the complexity of the Unity and the variety of possible tests, it is likely that physicists will not find comparative values in current publications for all commissioning work performed locally. Finally, given the novelty of the system, it can be argued that lack of experience or training would be a challenge during commissioning [7]; which can be alleviated in part with clear guidance or methodologies in publications.

The significance of our work is in the novel QA methods that describe alternative uses for vendor supplied phantoms and the use of simple, in-house developed phantoms and software. This work aims to provide straightforward methodologies that can be employed by most clinics for performing these tests, as well as comparative baseline results for Unity users. Additionally, we provide alternate methods for independently verifying vendor measurements. Adaptations of an Elekta supplied phantom enabled independent QA of the gantry angle and MV isocentre size. Development of in-house phantoms was required for measurements of beam attenuation, due to the anterior imaging coil, and end-to-end (E2E) testing. Difficulties were encountered with commercial equipment when measuring beam quality and output, and when performing IMRT commissioning, that required adaptations to standard methodologies and as such are also presented below. During installation there was limited time to facilitate customer selected measurements prior to magnet ramp up; although a spontaneous quench and planned ramp down events enabled selected commissioning tests to be repeated with B = 0 T. For brevity, more common commissioning measurements are not provided in this work and will be published subsequently. These include mechanical behaviour of the system, MR-to-MV isocentre offset confirmation, relative dosimetry measurements, a thorough investigation of the treatment planning system (TPS), commissioning of the MR system, MLC characterisation [8], mutual interference of MR and MV systems and a radiation survey within the treatment room.

## Methods

### Planning system simulations

A thorough assessment of the TPS modelling of the Unity is out-of-scope for this work; however, it is used throughout this work to generate comparative values for select tests. At the time this work was conducted the commercial TPS provided with the Elekta Unity MRL was Monaco v5.40. It uses a GPU Monte Carlo dose (GPUMCD) algorithm for fast calculations in the presence of a static 1.5 T magnetic field [9, 10]. Dose calculations are performed on voxelized models of volumes, with relative electron density (RED) assigned based on a CT specific, CT number-to-RED table. For calculations on MR datasets, RED is assigned per structure with user specified values; typically, the mean RED for a structure is calculated from a reference CT scan. REDs are mapped to chemical composition using patient, phantom or couch material look-up tables. Users can specify a dose calculation grid resolution and statistical uncertainty to control the accuracy of calculations. For plan adaption two workflows are available, adapt-to-position (ATP) or adapt-to-shape (ATS). ATP involves repositioning of pre-treatment (reference) plan isocentre, based on the rigid registration of that plan and image dataset with a daily MR image [11]. The pre-treatment plan can be recalculated or reoptimized on the reference dataset to reproduce or improve target dose coverage. ATS allows for plan adaption based on anatomical changes as shown on the daily MR image. Contours can be automatically deformed to match the daily anatomy, with optional user-based adjustments. The reference plan is recalculated or reoptimized using reference plan constraints [11]. For more information on the replanning options the reader is referred to other works [9–11].

### Gantry angle

**Fig. 2:**
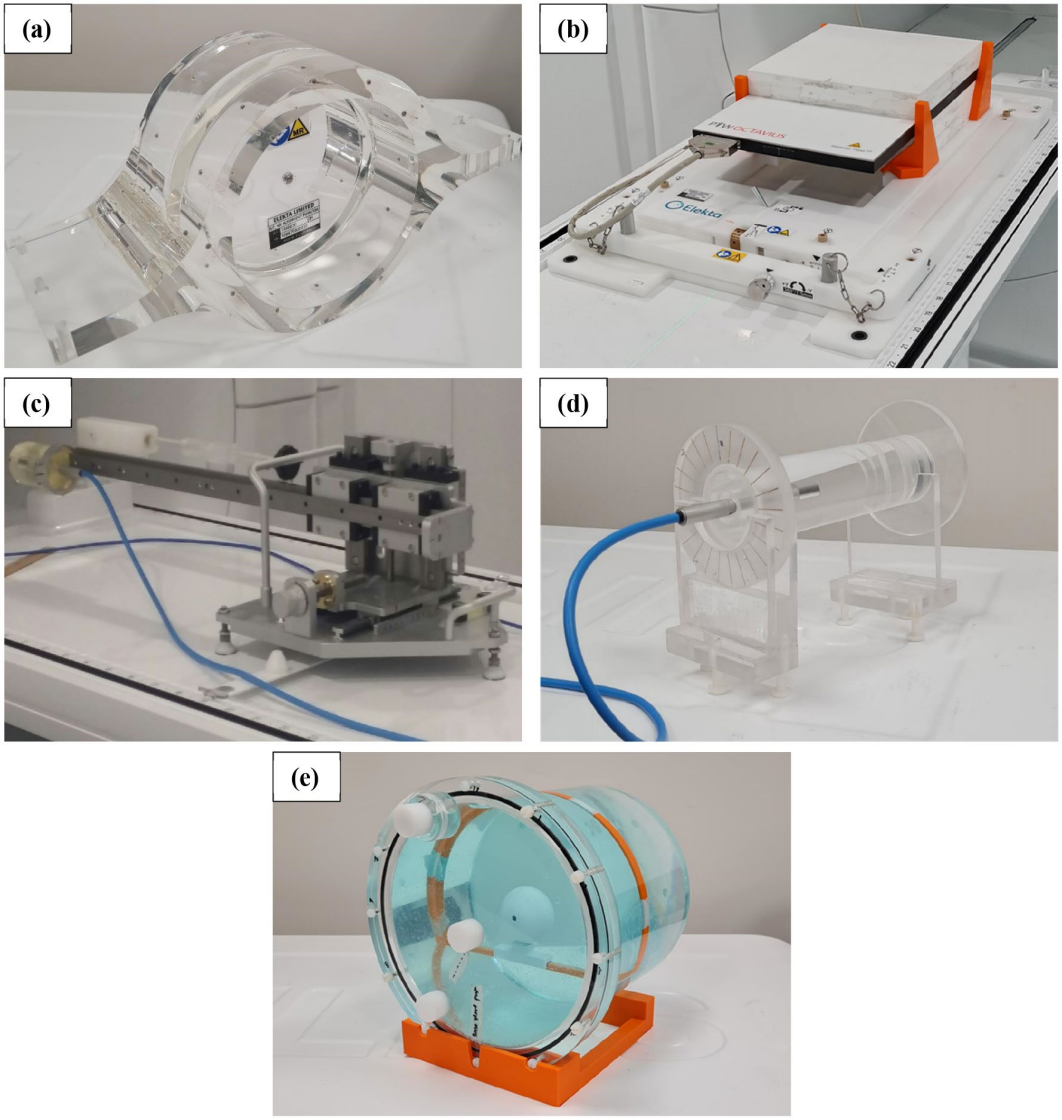
Images of the Elekta supplied (**a**) MV alignment phantom (**b**) QA Platform with the PTW Octavius 1500^MR^ array and solid water stack on top (**c**) cryostat characterisation tool, as well as the in-house developed (**d**) cylindrical phantom used for rotational output measurements and (**e**) acrylic phantom used for E2E workflows

Due to the construction of the gantry ring, the use of an inclinometer to determine gantry angle is limited to 270.0° and 240.0° [5]. As part of routine QA for conventional linacs it is common to determine gantry angle accuracy for multiple positions and to assess the reproducibility of gantry rotation. Previous publications report on the use of spoke films to assess relative beam angles [3, 5]. Additionally, a unique phantom design has been described for gantry angle QA on the Elekta Unity [12]. However, in the absence of such a phantom, and as an alternate to spoke film measurements, we propose the use of vendor supplied equipment, Fig. 2a and b, that can be adapted to investigate gantry angle positioning.

The vendor supplied MV alignment phantom (Elekta, Stockholm, Sweden), shown in Fig. 2a, facilitates determination of several machine mechanical characteristics. This cylindrical, acrylic phantom contains a centrally located, 10.0 mm diameter ballbearing, with two arrays of twelve 4.0 mm ballbearings, radially arranged at 30.0° intervals, and offset by ± 3.5 cm in the Y-direction. With this phantom positioned on the Elekta supplied QA platform (see Fig. 2b), MVI images of the phantom were obtained at the cardinal gantry angles and analysed with vendor software, to determine offsets from isocentre. The phantom was then repositioned using the QA platform with X, Y and Z vernier adjustments. Additional MVI images were used to confirm the phantom position until offsets were less than 0.2 mm and 0.2°. The phantom was then shifted ± 3.5 cm, so that the centre of a given array of ballbearings was nominally at the isocentre.

MVI images of the phantom were obtained with the gantry angle varying from 0.0° (G0) to 360.0° in 30.0° increments using a 10.0 × 10.0 cm^2^ field, 50 MU. Each image showed diametrically opposed ballbearings at the centre, as well as projections of off axis ballbearings at the peripheries. A self-levelling laser was used to verify that ballbearings at twelve and six o’clock were aligned vertically within the phantom. Visual inspection of the images from each angle was performed to confirm that diametrically opposed ballbearings were eclipsed, producing an image of a single ballbearing. For all measured angles, the separation between ballbearings at the image peripheries was also assessed for consistency between rotations. Additional MVI images were obtained with the gantry rotated ± 1.0° from vertical, in increments of ± 0.1°, to assess the resolution of the central and peripheral ballbearings. For these projections, gantry angle change was measured with a digital Clinotronic PLUS inclinometer (Wyler AG, Winterthur, Switzerland) between each rotation to confirm the relative shift from G0. This was placed at a fixed position on a convenient surface of the gantry ring.

In addition to visual inspection of the images, pixel value profiles were extracted using ImageJ v1.53a (NIH, Bethesda, MD, USA) [13]. Profiles through the centre of all ballbearings, in each MVI image, were obtained for all delivered gantry angles and compared to that for the G0 image. For the deliberate mis-alignment of the gantry from the vertical, it would be expected that pixel profiles shapes would vary from that of the G0 image. Additionally, as a first-order approach, profiles taken through the images at 30.0° increments could be assessed against those with the deliberate sub-degree offsets to confirm rotational accuracy within published tolerances [3, 5].

### MV isocentre diameter

During device acceptance tests (DAT) an Elekta supplied cryostat characterisation tool (CCT), see Fig. 2c, is used with the MVI to obtain a series of images of a ballbearing for MV isocentre determination. These images are forwarded to Elekta for analysis using radiation isocentre tool (RIT) software v6.6.64 (Radiological Imaging Technologies, Colorado Springs, Colorado, USA). This is not optimal given the inherent turn-around time for results. Alternatively, Snyder et al*.* [4] described the use of the MV alignment phantom, Fig. 2a, to obtain MVI images of the centrally located ballbearing; however, image analysis again relied on access to RIT software. Furthermore, for their work, lateral angles had to be avoided due to limitations of the ballbearing edge detection in RIT [4]. Another option available to users for analysing such images is with the Elekta supplied AQUA™ software, which contains an isocentre measurement test for the MRL. Licences for this software are often provided with purchase of the Unity system; however, the use of the test suffers from the same angle restriction as that of Snyder et al*.* [4]. In our work, we demonstrate the use of the same phantom to determine the MV isocentre using in-house software analysis and MV imaging from a broader range of gantry angles. A comparison of results for this methodology against those of Elekta and Snyder et al*.* [4] is provided.

For our determination of the MV isocentre size, the MV alignment phantom (Fig. 2a) was mounted on the QA Platform and aligned to the isocentre using the methodology described previously. Note that the vendor alignment software for positioning the phantom at isocentre does not report an isocentre size. With the centre of the phantom at isocentre, the central ballbearing was projected onto the MVI using 5.0 × 5.7 cm^2^_,_ 50 MU, fields every 10.0° for gantry angles ranging from 0.0° to 360.0°. In contrast to the methodology used by Snyder et al*.* [4] and Elekta, gantry angles of 10.0° and 20.0° were avoided (due to the presence of the cryostat cross-over pipe that provides electrical connection between the split coils), as well as 60.0° and 300.0° (due to distortion from the couch edges through the centre of the ballbearing image). Images with the gantry rotating in both directions were obtained in a single sequence, for a total of sixty-nine projections. The images were exported from the MVI computer and analysed with in-house MATLAB® code (MathWorks, Natick, Massachusetts, USA). Vertical and horizontal pixel intensity profiles were extracted from each MVI image to determine the ballbearing position.

When imaging from 130.0° to 50.0° and 310.0° to 230.0°, the phantom, QA platform and couch edges created distortion in the images. To minimize this effect, horizontal background pixel profiles just above and below the ballbearing were acquired for a given projection, averaged, and then subtracted from the horizontal profile through the ballbearing. The FWHM of the ballbearing profile was then more accurately determined from the result. The centre pixel of the FWHM peak was compared against the MVI central pixel, at each projection, to determine the MV isocentre. Note that the coincidence of the central pixel to the isocentre (at multiple angles) was performed by Elekta prior to DAT, the method for which is outside the scope of this work. However, it was accounted for when presenting values. Routine use of this technique requires accurate and consistent alignment of the beam line with the BLD and MVI panel (at multiple angles), and this should be ensured by clinics before adopting this method; however, is not expected to vary significantly once established [5]. For context, on our system the maximum variation of the central pixel (from G0) since commissioning is less than 0.7 pixels, as determined using results from an in-built test within AQUA™.

To compare with Elekta results acquired during DAT, measurement of the isocentre size using the in-house method was also performed with the CCT. Furthermore, the method of Snyder et al*.* [4] was replicated with the MV alignment phantom. Isocentre sizes were compared between phantoms, beam sequences and magnetic field environments. During the in-house physics validation measurements, spoke shot films were obtained with Gafchromic™ RTQA2 (Ashland ISP Advanced Materials, NJ, USA) film following the methodology proposed by previous researchers, wherein copper rings are introduced [3, 14]. The spoke shot dose distribution under the rings is less influenced by the interaction of electrons with the magnetic field, improving the precision of the isocentre determination in the X–Z plane. These results are also provided for comparison.

### Anterior coil attenuation

For all treatments on the Unity, the anterior imaging coil is present above the patient and should be fully characterised in terms of it is dosimetric impact on treatment beams [5, 6]. However, to our knowledge no work has been published on a specific method for this. Additionally, this is not performed by Elekta during DAT for individual installations, with a factory default value for the RED applied in the TPS.

Attenuation of the anterior imaging coil, as function of gantry angle, was determined with a PTW 30013 Farmer chamber within an in-house, cylindrical, water-filled phantom of diameter 6.0 cm and length 15.0 cm. The long axis of the chamber was coincident with the central axis of the cylinder, see Fig. 2d, with the Farmer threaded section and tapped cylindrical hole providing a waterproof seal. The cylindrical section of the phantom is free to rotate on two height-adjustable stands and a scale at one end facilitates angular positioning in 15.0° increments.

The phantom was positioned in the bore with the chamber reference point at the isocentre. To realise this, A-P and L-R MVI images were used to determine necessary lateral and height adjustments. Chamber readings were obtained using a 5.0 × 5.0 cm^2^ field, 100 MU, for 15.0° gantry angle increments from 75.0° to 285.0° with and without the coil present. For each gantry angle, the cylindrical phantom was also rotated to maintain the same orientation of the chamber with respect to the beam. Readings, with and without the coil, were compared to determine attenuation at each angle. The experimental arrangement was simulated in Monaco using a 6.0 cm diameter, 15.0 cm long, cylindrical structure for the phantom, which was assigned a forced RED of 1.000. Isocentre position for calculations was centred in the X–Z plane of this structure, using virtual couch shifts, and longitudinally adjusted to match the position of the chamber reference point. For measurement and calculation, the coil height was set such that the bottom of the coil was 26.0 cm above the couch. A 0.1 cm dose grid, statistical uncertainty of 0.25% per control point and the patient look-up table were used in Monaco to calculate the dose-to-medium.

### X-ray beam quality

The beam quality specifier for the Elekta Unity MRL is the TPR_20,10_ [6, 15–17], consistent with the recommendations of the TRS-398 protocol, and because of its insensitivity to the magnetic field [6, 16, 17]. Due to the difficulties with measuring PDDs, a direct measurement of TPR_20,10_ is preferred and because of the cryostat, slight angular dependence of beam quality could be expected. With beam quality dependent factors for reference output, a determination of this angular dependence, or at minimum an assessment of the change in these factors with beam quality, should be evaluated. Previous Monte Carlo simulations [18] have suggested that a TPR_20,10_ change of 0.679 (6 MV linac) to 0.703 (7 MV MRL) did not influence beam quality dependent factors for the PTW 30013 Farmer by more than 0.5%; however, it was felt that confirmation with measurement was warranted.

Furthermore, profile shape is known to be more sensitive to beam quality changes than TPR or PDD metrics. Profiles are generally acquired in water, using a 3D scanning water tank; however, such a device is not available to all Unity clinics and, even if available, is cumbersome to use routinely. Hence, dose maps were acquired on the Unity using the Octavius 1500^MR^ array (PTW, Freiburg, Germany) to assess the beam shape.

For TPR_20,10_ measurements the previously mentioned Farmer chamber was inserted in the PTW 1D water tank. The chamber was aligned parallel to the Y-axis, with the reference point at the isocentre in the X–Y plane using A-P/L-R MVI images. Due to the size of the water tank, the chamber was lowered 4.0 cm below isocentre (SCD 147.5 cm) to facilitate measurements at a depth of 20.0 cm in water. Measurements at 10.0 cm (SSD 137.5 cm) and 20.0 cm depths (SSD 127.5 cm) were obtained using a 9.7 × 9.7 cm^2^ (10.0 × 10.0 cm^2^ at the depth of the chamber), 100 MU field, G0. TPR_20,10_ was derived from the ratio of the average readings (n = 3) for each depth, with repeat measurements performed across several days.

To investigate the impact of the cryostat on beam quality, TPR_20,10_ measurements were also performed from G90 using a 10.0 × 10.0 cm^2^ 100 MU field, with the chamber at isocentre. The acrylic tank wall was measured to have a water equivalent thickness of 1.2 cm, therefore for these measurements the chamber was set 8.8 and 18.8 cm from the inner surface of the wall. This was achieved using a 3D-printed 8.8 cm indexer (confirmed to be the correct length with a calliper) to position the chamber reference point at 10.0 cm of water equivalent depth. A-P/L-R images were used to determine required shifts to position the water tank such that the chamber was at isocentre. For readings at 20.0 cm, A-P images were used to shift the chamber 10.0 cm laterally (− X) and reposition the tank, so the chamber remained at isocentre.

For profile measurements, the QA Platform was placed on the Unity couch with the patient foam mattress removed. Using four in-house 3D-printed holders, 6.5 cm of solid water (RW3, PTW, Freiburg, Germany) was positioned centrally on the QA Platform. Printing material used was polylactic acid (PLA). With the 1500^MR^ array on top of the solid water, see Fig. 2b, the effective plane of measurement of the array was at isocentric height. The orientation of the array with respect to isocentre was checked using a 22.0 × 22.0 cm^2^ G0 MVI image, with an in-house aluminium “ruler” aligned on the X-axis of the array. The ruler is 2.5 cm wide and 30.0 cm in length, with thirteen machined 3.0 mm holes spaced 2.0 cm apart along its length. Misalignment of the ruler/array with the centre of the MVI image was determined using the MVI measurement tool, and array rotation was assessed using the horizontal markers on the ruler against the 1.0 cm MVI digital grid. Necessary position adjustments were identified and applied using the X and Y verniers on the QA platform. Following adjustments, additional MVI images were obtained to confirm array offsets and rotations from isocentre were negligible. With the array correctly aligned, the ruler was removed and 4.2 cm of solid water was added (0.8 cm intrinsic build up), so that the detector plane was at the calibration depth of 5.0 cm. The array was then calibrated with a 10.0 × 10.0 cm^2^ field, 100 MU, delivered from G0. Finally, a 22.0 × 22.0 cm^2^, G0 field with 100 MU was delivered to the array to determine dose maps at 5.0 cm depth.

The two measurement geometries described above were simulated in the Monaco v5.40 TPS. For the TPR_20,10_ simulations, two separate datasets were used where heights of the external contours produced the SSDs of the measurement geometries (137.5 and 127.5 cm). Remaining dimensions of the contours were set to 20.0 cm to decrease calculation time whilst maintaining full scatter conditions. For profile calculations, a 30.0 × 30.0 × 19.0 cm^3^ region was contoured and set as the external structure. All three external structures were assigned an RED of 1.000. TPS calculations were performed with a 0.2 cm dose grid, 0.1% statistical uncertainty per control point, the phantom look-up table and dose deposition to medium. With these settings, the statistical uncertainty at the regions of interest was less than 0.15%. For profiles, extracted from 5.0 cm depth in the TPS, gamma analysis between calculated and measured dose maps was performed with 2.0% local dose, 2.0 mm distance-to-agreement (DTA), with dose suppression below 10.0%, as per routine clinical practice.

### Reference dosimetry

During DAT, on the recommendation of Elekta, the MRL was calibrated to give 1.000 Gy per 100 MU to isocentre, at 5.0 cm depth in water, for a 10.0 × 10.0 cm^2^ field, G90. Measuring from G90 is the preferred methodology due to the constancy of the helium fill at this angle compared to acute anterior angles, like G0, where the output may vary depending on the level of helium. The choice of calibration depth was based on advice provided by Elekta to extend magnetron life; however, other users report a calibration depth of 10.0 cm to optimise output [4], thereby reducing treatment times. The PTW 1D water tank (PTW, Freiburg, Germany) is often used for output determination, as well as the dosimetry protocol proposed by van Asselen et al. [16]. When using the current version of the PTW 1D tank, a direct measurement of the output at isocentre beneath 5.0 cm of water cannot be achieved at G90, due to collision of the tank with gantry covers. Future versions of the PTW tank will potentially address this issue; however, at present the measuring technique using this tank needs to be adapted to follow the Elekta recommendation. When using plastic phantoms, air gaps around the chamber give rise to the electron return effect (ERE), thereby increasing measurement uncertainty [17, 19] and as such their use is typically avoided.

For reference dosimetry measurements on this system, a PTW 30013 Farmer chamber was placed in the PTW 1D water tank, with the chamber reference point positioned at isocentre using MVI images. Readings were acquired using 10.0 ×  10.0 cm^2^, 100 MU, G0 fields, with the chamber reference point at 5.0 cm (SSD 138.5 cm) and 10.0 cm (SSD 133.5 cm) depths, to derive a TPR_10,5_. Next, to determine the output from G90 required the tank to be shifted 6.2 cm laterally (− X direction) so the chamber was at isocentre, 10.0 cm depth, whilst accounting for the water equivalent thickness of the tank wall. Chamber readings with the same field, now delivered from G90, were acquired at this new position and corrected to 5.0 cm, using the G0 TPR_10,5_, to give the machine output. Chamber influence quantities for temperature and pressure, polarity and recombination, and a published magnetic field correction factor, ${k}_{{B}_{\parallel },Q}$ of 0.992 [16] were applied to the readings at 10.0 cm depth, G90. A non-uniformity correction factor [20] for the FFF beam was not applied given the relative flatness of the profiles at 10.0 cm depth over the dimensions of the chamber sensitive region [17]. Measurements were repeated across several weeks to assess reproducibility of this method and the stability of machine output. Routinely, measuring the output from G90 using the methodology described above can be cumbersome. It is known from the cryostat attenuation of our machine, measured during DAT, that the output at isocentre for G0 is 0.5% higher than from G90. Hence for routine QC, and as an independent check on the methodology adopted above, the output of the machine from G0 was determined, where a direct measurement at isocentre beneath 5.0 cm of water was readily achieved.

The use of published magnetic field correction factors at 10.0 cm [16, 17] for a calibration depth of 5.0 cm can be questioned. Additionally, it is beneficial for users to determine these factors for their specific chambers if possible [16]. Following a ramp down event, detector dependent magnetic field correction factors (${k}_{{B}_{\parallel },M,Q}$ [16]) for two PTW 30013 chambers (S/N 10765 and 11298) were determined following the formalism by van Asselen et al*.* [16], at the two calibration depths. Additional measurements were made in the 0 T environment, with the previously described G0 fields, to investigate the depth dependence of this factor. Since this work was performed to confirm applicability of using published factors (10.0 cm) at a depth of 5.0 cm, no reference chamber was used for these measurement [21], increasing the uncertainty in the determined factors.

### IMRT commissioning

As part of the Elekta beam validation procedure, nine vendor IMRT plans, based on AAPM TG-119 [22] guidance, were imported to the Monaco treatment planning system (TPS) and delivered on the Unity system. Elekta recommends gamma criteria of 3.0% (global dose difference) and 3.0 mm DTA during beam validation, with the ArcCheck®-MR device (Sun Nuclear, Melbourne, Fl, USA). This device was not available during commissioning and in its absence the Octavius 1500^MR^ and Gafchromic™ EBT3/EBT-XD film (Ashland ISP Advanced Materials, NJ, USA) were utilized. The methodology for planning system calculations and the various measurement techniques are provided below.

#### Planning system calculation

A 30.0 × 30.0 × 19.0 cm^3^ solid water stack was scanned on a Toshiba Aquilion CT using 1.0 mm slices. The CT dataset was imported into the QA clinic in Monaco and contoured as an external patient structure. The RED of the structure was forced to 1.000 and the MRL couch structures were added, excluding the 1.0 cm foam mattress. All plans were calculated on this QA dataset.

Following clinical practice, TPS calculations for Elekta plans used a statistical uncertainty of 3.0% per control point and a 0.3 cm dose grid. For stereotactic IMRT plans, a 0.2 cm dose grid and 3.0% statistical uncertainty per control point were used. For both settings, the overall calculated dose uncertainty was 1.0% or lower. The phantom look-up table, with dose deposition to the local medium, was selected. For all plans, calculation times were less than 2.0 min.

#### Simple segment shape check

To confirm correct MLC shaping, the segments of each stereotactic plan were delivered to the MVI panel at their respective planned gantry angles. Due to the restricted imaging area of the panel (22.0 × 9.5 cm^2^), this was not performed with the Elekta plans. The size, shape and position of the individual segments were visually compared to corresponding Monaco segments.

#### Perpendicular delivery

At our centre, film is considered the gold-standard for IMRT patient specific quality assurance (PSQA) of treatment plans on conventional linacs, with other detectors like the Octavius 1500^MR^ array being benchmarked against film during commissioning. To this end, the nine vendor IMRT plans and two in-house developed stereotactic plans were delivered at G0 to both film (Gafchromic EBT3 or EBT-XD, Ashland ISP Advanced Materials, NJ, USA) and the array. Figure 2b shows the setup of the Octavius 1500^MR^ array on the QA platform for IMRT plan verification measurements. Array setup and calibration was performed with the same methodology as described above with profile measurements. Fields for the eleven IMRT plans were delivered perpendicularly to the array, beam-by-beam, and resulting dose maps were recorded. Comparisons of beam-by-beam dose maps and a composite dose map were made to those from the TPS. Gamma analysis was performed with Verisoft v7.2 software (PTW, Freiburg, Germany) and criteria of 2.0% of local dose, 2.0 mm DTA and dose suppression below 10.0%.

Choice of film for measurements depended on dose per fraction; EBT3 film was used for doses less than approximately 8.0 Gy and EBT-XD was used above 8.0 Gy. The suitability of these film types at these dose levels has been investigated previously [23]. For film dosimetry, the QA Platform was placed on the couch with the foam mattress removed and 30.0 × 30.0 × 8.0 cm^3^ of solid water was placed on top, with 3D-printed supports (Fig. 2b) that nominally centre the solid water at isocentre in the X–Y plane. With this setup, the upper surface of the solid water was at isocentre height. Crosslines marked through the centre of the solid water were used to position the aluminium ruler and aid with aligning the phantom and QA platform to isocentre, using the methodology described previously. After phantom alignment, film calibration was performed on the Unity using a geometric dose progression with five 4.0 × 2.0 cm^2^ strips of film, to encompass the maximum delivered dose for all plans [24]. The calibration was performed using 10.0 × 10.0 cm^2^ G0 fields, film at 5.0 cm depth. For plan QA, films were centred on the solid water stack at 5.0 cm depth, and crosslines on the solid water, indicating X and Y axes, were used to mark the film orientation. Fields for all plans were delivered compositely from G0 to individual films. Films were scanned using an Epson 10000XL scanner at 72.0 dpi and scanner corrections were applied for the Lateral Response Artefact [25]. Comparisons to the planning system were made using FilmQA™ Pro v5.0 software (Ashland ISP Advanced Materials, NJ, USA) and triple channel analysis [24]. Comparison of TPS and measured dose distributions was performed using gamma analysis with 2.0% of global dose, 2.0 mm DTA and dose suppression below 10.0%.

#### True composite delivery

Delivering plans perpendicularly from G0 obviously does not simulate the treatment geometry. Consequently all plans were delivered at planned gantry angles to a coronal film within a solid water block. Again, depending on dose level, EBT3 or EBT-XD films were used. Prior to measurement, optimal plan dependent depth for the film was identified using Monaco. Choice of depth primarily depended on beam geometry, beam weighting and the resulting dose distribution; however, typically a coronal slice was chosen through the centre of beam convergence and steep dose gradients were avoided. Film dosimetry was performed using a solid water phantom 30.0 × 30.0 × 19.0 cm^3^, placed on the couch (mattress removed) and aligned to the X and Y axes using the aluminium bar and MVI images as outlined above. The anterior coil was excluded during measurements, and the posterior coil was included to replicate the TPS calculations. Film calibration and analysis were performed as discussed previously. To investigate the potential impact of the electron return effect (ERE) on the film dose, due to the presence of air gaps between the film and solid water, measurements were repeated with the calibration and plan delivery films sprayed with water [26].

### End-to-end

Elekta provides phantoms for ATP and ATS E2E testing on the Unity during physics validation; however, a clinic may want to perform their own measurements for routine QA and anatomical treatment site commissioning. Purchasing specific phantoms for individual site development can be expensive and furthermore, a centre may not have ready access to the commercial, MR compatible, phantoms for adaptive radiotherapy E2E testing used during the work with Elekta. The use of in-house developed 3D-printed phantoms can alleviate these issues. 3D-printed materials have been extensively used in conventional radiotherapy applications [27–29] and their use in MR-guided radiotherapy (MRgRT) systems has seen much development [30, 31]. An in-house designed phantom, with 3D-printed components, was used for E2E measurements for baseline comparisons on the Unity in this work.

This in-house phantom had components 3D-printed at the Royal Brisbane and Women’s Hospital, Australia. The phantom comprised of a hollow acrylic cylinder (20.0 cm length, 22.0 cm diameter) containing a 3D-printed frame and a platform on which 3D-printed tumour surrogates could be mounted, Fig. 2e. All printing material was PLA and had a nominal RED of 1.050. Surrogates were hollow, hemi-spherical and half-cylindrical shells with known internal radii and shell thickness. The hemispheres/half-cylinders could be secured together with film in-between to facilitate dosimetric measurements and were printed with holes for filling the hollow sections with MR-visible material. The platform had multiple points in which the surrogates could be inserted at known offsets for testing of either ATP or ATS workflows. A thorough characterisation of the phantom and printing materials was performed on the system; however, is beyond the scope of the Unity commissioning work.

The E2E water-filled phantom containing a 2.5 cm radius spherical 3D-printed surrogate was scanned on a Toshiba Aquilion CT with 2.0 mm slices and the image dataset was imported into Monaco. Contours of the phantom and its components were defined and forced REDs were applied to the respective mean values as calculated by the TPS. Calculated REDs agreed within 0.6% of the nominal value for the 3D-printed components. The water filled sections of the hemispheres were contoured together, designated as the target and set to enable automatic deformable registration. A margin expansion of 1.5 mm was applied to the target, to generate a contour for the outer surface of the spherical shell. Dose-to-medium within the phantom was calculated using a seven beam Step-and-Shoot IMRT (SSIMRT) plan. A 0.3 cm dose grid and 3.0% statistical uncertainty per control point was used for calculations. Inclusion of the anterior coil in the E2E workflow required the use of the patient look-up table, and as such phantom components were mapped to tissue materials. With these parameters, the statistical uncertainty was less than 0.8% at the film location and the optimization time was 140.9 s.

For delivery, the phantom was positioned on the Unity couch with 3D-printed frames attached to an accessory fixation lock bar to locate the phantom. The spherical surrogate was inserted in the platform, offset from the centre (0.5 cm X and 1.0 cm Y), with a piece of Gafchromic EBT3 film set between the hemispheres. A 2.0 min, T2-weighted MR-image was acquired, registered to the CT dataset and an ATP plan was calculated using segment shape optimization (SSO) and segment weight optimization (SWO), with the aim of reproducing goal dose [11]. Objective function parameters were not altered from their default values and the resulting recalculation time for the ATP plan was 47.8 s. The newly generated plan was then delivered to the phantom. Following this the spherical surrogate was replaced with a cylindrical one, at the same offset as above, of internal radius 2.5 cm, length of 5.0 cm and a film strip positioned along its longitudinal axis. Again, a 2.0 min T2-weighted MR scan was acquired and registered to the CT dataset; however, now the ATS workflow was used. The auto-deformed target contour was visually checked for accuracy and manually adjusted as necessary, following clinical workflow. The ATS plan was generated from fluence with five iterations, as per the clinical default. With these settings, and the same calculation settings as for the reference plan, optimization time for the ATS plan was 149.9 s. Each film was compared to the planning system using FilmQA™ pro and gamma analysis with 2.0% global dose, 2.0 mm DTA and dose suppression below 10.0%.

## Results

### Gantry angle

**Fig. 3:**
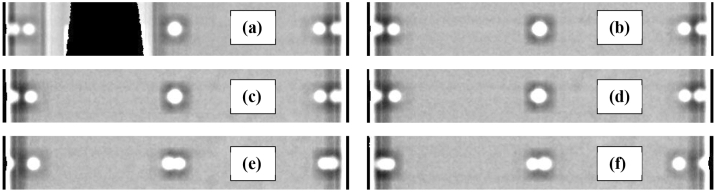
MVI images of the superior outer ring of ballbearings on the MV alignment phantom. Gantry angles of (**a**) 270°, (**b**) 0.0°, (**c**) 0.2°, (**d**) 0.3°, (**e**) 1.0° and (**f**) 359.0° are presented

**Fig. 4:**
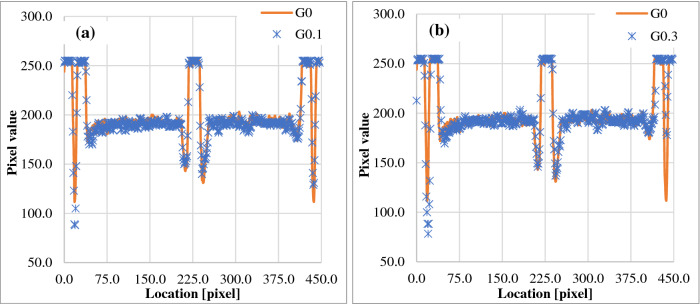
Comparison of profiles taken left to right from MVI images for the ballbearings at (**a**) G0 and G0.1 and (**b**) G0 and G0.3

Representative MVI images of the gantry angle measurements are shown in Fig. 3. Images have been auto enhanced within MVI to highlight pixel value gradients. With a set gantry angle of 270.0° the inclinometer measured an angle of 270.02° and the set angular shifts from 0.0° were also confirmed to be within ± 0.02°. The attenuation effect of the acrylic phantom is apparent in the 270.0° image (Fig. 3a). Images every 30.0° from gantry zero were indiscernible from the G0 image, Fig. 3b, except for G270 and G90 where the thicker acrylic component of the phantom was being imaged (see Fig. 2a). Diametrically opposite ballbearings were eclipsed for those images. Additionally, pixel intensity profiles for three angles are given in Fig. 4 (G0, G0.1 and G0.3).

### MV isocentre diameter

Results for the various isocentre measurement techniques are presented in Table 1. All techniques showed that the isocentre was within tolerance (≤ 1.00 mm [3]).

**Table 1:** MV isocentre results for the various methodologies, analyses and magnetic field environments

Equipment and method	Magnetic field [T]	Isocentre diameter [mm]
CCT + RIT analysis	1.5	0.45
MV alignment + RIT analysis	1.5	0.42
CCT + in-house analysis	1.5	0.34
MV alignment + in-house analysis	1.5	0.38
MV alignment + in-house analysis	0.0	0.32
Spoke shot with copper ring	1.5	0.36
Spoke shot with copper ring	0.0	0.24
Spoke shot without copper ring	0.0	0.28

The use of the CCT with the RIT analysis was performed by Elekta during DAT and the use of the MV alignment phantom and RIT analysis is performed during the physics validation stage, and is consistent with that discussed by Snyder et al*.* [4]

### Anterior coil attenuation

For the range of angles investigated, measured and calculated coil attenuation values as a function of gantry angle are shown in Table 2. The average measured attenuation was (0.6 ± 0.1)%, compared to the calculated average (0.8 ± 0.1)%.

**Table 2:** Measured and calculated anterior coil attenuation as a function of gantry angle

Gantry angle [°]	Measured attenuation [%]	Monaco attenuation [%]
75.0	0.0	0.0
60.0	0.6	0.8
45.0	0.6	0.9
30.0	0.5	1.0
0.0	0.5	0.7
345.0	0.4	0.8
330.0	0.7	0.9
315.0	0.6	0.8
300.0	0.6	0.7
285.0	0.0	0.0

### X-ray beam quality

**Fig. 5:**
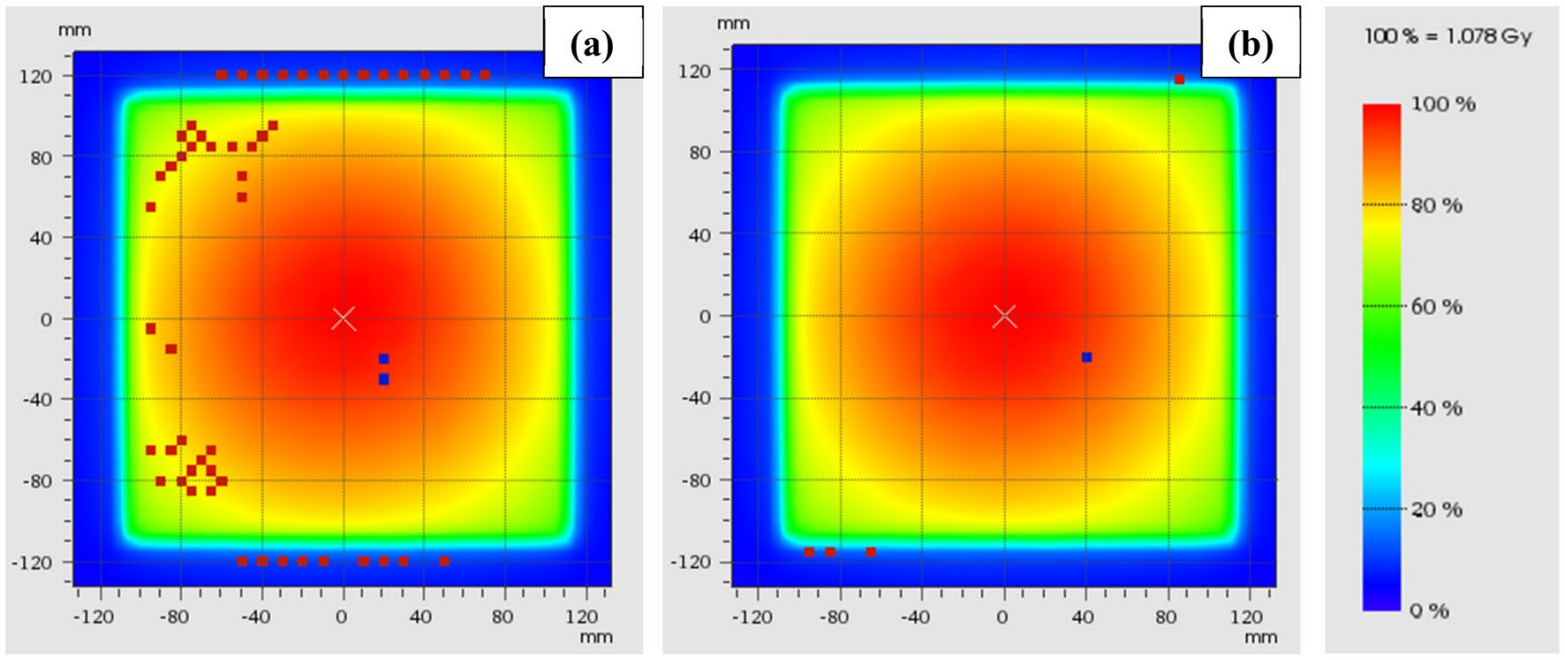
Dose maps for 22.0 × 22.0 cm^2^ fields measured on the Octavius 1500^MR^ array and compared to TPS calculations in Verisoft using gamma analysis at 2.0%, 2.0 mm criteria for (**a**) the original commissioning magnetron and (**b**) with the new magnetron. Regions of hot/cold failure are indicated by the red/blue dots, respectively

The measured TPR_20,10_ in the B = 1.5 T environment was 0.705 ± 0.001 (n = 4) and for the original magnetron with B = 0 T, the TPR_20,10_ was 0.703 (n = 1). Planning system TPR_20,10_ was calculated as 0.702. Following a magnetron replacement, the TPR_20,10_ from G0 was measured as 0.703 ± 0.001 (n = 5) and from G90 was 0.703 (n = 1). A dose map comparison of the measured beam with the original magnetron/TPR_20,10_ to the TPS is shown in Fig. 5a (95.3% gamma pass rate, at 2.0%, 2.0 mm criteria) and similarly for the new magnetron/TPR_20,10_ in Fig. 5b (99.6% gamma pass rate).

### Reference dosimetry

From G0, measured output at isocentre was 1.002 ± 0.004 Gy per 100 MU (n = 7) at depth 5.0 cm in water, and TPR_10,5_ was 0.858 ± 0.001 (n = 5). From G90, output was 1.000 ± 0.002 Gy per 100 MU (n = 7) at the isocentre beneath 5.0 cm of water, and in the B = 0 T environment, the output at G0 was measured as 1.020 Gy per 100 MU (n = 2). At 5.0 cm depth, G0, the detector magnetic field correction factors, ${k}_{{B}_{\parallel },M,Q}$, were 0.995 (S/N 10765) and 0.996 (S/N 11298 ) for n = 1. Similarly, with the chambers at 10.0 cm depth, ${k}_{{B}_{\parallel },M,Q}$ factors were 0.999 for both. After applying the dose conversion factor ${c}_{\tilde{B }}$, the values for the combined m agnetic field correction factors ${k}_{{B}_{\parallel },Q}$ were 0.990 (S/N 10765) and 0.991 (S/N 11298) at 5.0 cm and 0.994 for both chambers at 10.0 cm.

### IMRT commissioning

**Fig. 6:**
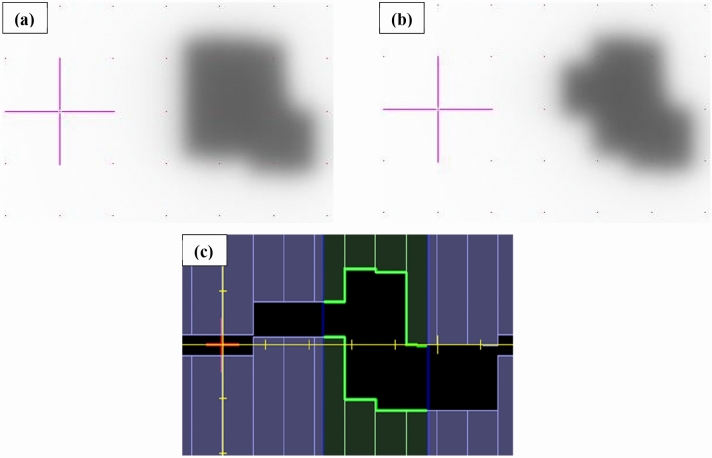
MVI images of segments for one of the in-house developed stereo plans. (**a**) Shows one delivered segment with the guard leaf error present and (**b**) shows the same segment with the error removed, matching that which was planned as indicated by (**c**) the beams-eye-view from Monaco TPS. All scales are in centimetres

A representative segment from one of the in-house stereotactic plans delivered to the MVI panel is shown in Fig. 6. Comparison of this MVI image (and similar) to the TPS segment, Fig. 6c, revealed a discrepancy between delivered MLC shapes and those calculated in the planning system, which ultimately was caused by erroneous guard leaf behaviour. Results from IMRT commissioning are shown in Tables 3, 4 and 5 for both pre- and post- guard leaf fix. Values in the tables are for dry film; however, when the patient and calibration films were sprayed with water, no statistically significant variation in the gamma results were noted. This was confirmed for multiple deliveries (n = 11) across several film batches.

**Table 3:** Gamma results for perpendicular deliveries to the 1500^MR^ array of the Elekta supplied TG119 plans and the two in-house developed stereotactic plans

Plan	Pre-fix		Post-fix	
	2%, 2 mm	3%, 3 mm	2%, 2 mm	3%, 3 mm
Abdomen	99.2	100.0	97.9	100.0
Head and neck	94.2	100.0	95.8	100.0
Lung	96.4	100.0	96.1	100.0
Multi-target	77.8	96.2	95.6	99.8
Prostate	97.4	100.0	97.8	100.0
Prostate_2	84.8	93.1	99.6	100.0
Prostate_7fld	88.4	94.8	100.0	100.0
Prostate_9fld	90.9	98.2	99.5	100.0
Prostate_11fld	96.5	100.0	100.0	100.0
Stereo 1	94.0	98.5	100.0	100.0
Stereo 2	71.2	82.0	98.6	100.0

**Table 4:** Gamma results for perpendicular deliveries to film of the Elekta supplied TG119 plans and the two in-house developed stereotactic plans

Plan	Pre-fix		Post-fix	
	2%, 2 mm	3%, 3 mm	2%, 2 mm	3%, 3 mm
Abdomen	96.2	99.8	96.0	99.7
Head and neck	91.7	95.7	99.7	100.0
Lung	68.8	86.0	99.9	100.0
Multi-target	77.4	92.9	98.0	99.9
Prostate	97.0	99.9	99.2	100.0
Prostate_2	75.7	90.5	97.6	99.7
Prostate_7fld	92.7	96.1	95.7	99.8
Prostate_9fld	95.2	99.3	97.0	100.0
Prostate_11fld	98.9	100.0	98.2	99.8
Stereo 1	70.2	92.1	97.3	99.3
Stereo 2			95.2	95.2

**Table 5:** Gamma results for composite deliveries to film of the Elekta supplied TG119 plans and the two in-house developed stereotactic plans

Plan	Pre-fix		Post-fix	
	2%, 2 mm	3%, 3 mm	2%, 2 mm	3%, 3 mm
Abdomen	74.5	94.7	96.5	99.7
Head and neck	92.3	99.1	97.4	99.8
Lung	80.4	97.4	98.0	100.0
Multi-target	79.1	94.8	97.3	99.8
Prostate	97.9	99.9	95.5	99.5
Prostate_2	87.4	97.1	96.9	99.9
Prostate_7fld			95.1	99.3
Prostate_9fld	98.0	100.0	98.8	100.0
Prostate_11fld			99.5	100.0
Stereo 1	81.2	96.3	97.8	99.6
Stereo 2			96.0	98.7

### End-to-end

The ATP created plan, for an adaption of the reference CT to a daily MR with the tumour surrogate shifted, passed with an average of 99.3% across the film colour channels (2.0% global dose, 2.0 mm DTA gamma criteria, dose suppression below 10.0%). The ATS created plan, with an adaption of the reference CT to a daily MR of a cylindrical surrogate, passed with an average of 97.5% across the three colour channels.

## Discussion

The results for the gantry angle measurement, as well as the MVI images in Fig. 3, highlight the accuracy of the gantry angle positioning of the Unity system. Imaging the MV alignment phantom from G270 introduced image distortions, hence G0 was chosen as the baseline image for gantry angle reproducibility with this method. The ballbearing positions on the G0 image appear to coincide with those on the G270 image. The MVI images show that gantry angle offsets of ≥  0.3° (Fig. 3d) from the nominal angle can be easily resolved, particularly when observing the ballbearings at image peripheries. With offsets  ≤ 0.2° (Fig. 3c) images ar e not readily discernible from the nominal (no angular offset) image without further analysis of pixel intensity profiles. Obviously, visual inspection cannot be used to det ermine the absolute gantry angle; however, it is useful for determining if set positions are within the tolerance specified by Roberts et al*.* [3] (± 0.3°). Note that the mor e recent publication of Woodings et al*.* [5] suggests a specification of < 0.2° for gantry angle, which cannot be achieved with visual inspection. For all projections at the 30.0° intervals, the actual gantry angle appeared to match the set position within 0.3°, further highlighting the accuracy of the gantry rotation system.

From Fig. 4, symmetry in the G0 profiles, particularly for the regions between ballbearings at image peripheries, was apparent. This symmetry was also seen for profiles taken from the images at the 30.0° gantry angle intervals, excluding G90 and G270 where image distortion interfered with the analysis. In comparison, the profiles for G0.1 (Fig. 4a) show asymmetry at the peripheries, which highlights the deliberate angular offset in image acquisition. This was magnified for the G0.3 image (Fig. 4b), which is the gantry angle tolerance [3]. Notably, the comparison of profiles for the different gantry angles does not result in an absolute gantry angle, but rather a confirmation of reproducible position for routine QA. However, these results indicate a proof-of-concept for the use of this phantom, supplied to all Unity sites, for gantry angle QA. Further work is needed to assess how phantom set up affects results, with attention given to positional reproducibility and subsequent misalignment effects. Additionally, the process could be improved by imaging with the 3.5 cm longitudinal offset removed and confirming alignment of diametrically opposed ballbearings along the Y-axis on MVI images. An absolute gantry angle offset may also be able to be determined from the offset of ballbearing centres from the lateral pixel centre on the panel; however, correct rotational alignment of the MVI would need to be confirmed, such as that discussed by Woodings et al*.* [5].

The results presented in Table 1 highlight the benefit of the Unity’s gantry slip-ring over conventional C-arm systems for reducing the isocentre size [4]. The isocentre diameter as measured using the CCT with the commercially available RIT software was 0.45 mm. This is larger than other reported values [4] however still within vendor tolerance (1.00 mm). The isocentre as measured with the MV alignment phantom and Elekta recommended methodology was similar in magnitude. Using our in-house method with the CCT and the MV alignment phantom, isocentre diameters were 0.34 and 0.38 mm, respectively. These are again slightly larger than that reported by Snyder et al*.* [4] however are still within tolerance, with differences most likely due to variations in machine construction. In the 1.5 T environment, the spoke shot with copper technique, as suggested by Roberts et al*.* [3], reported an isocentre size in the X–Z plane comparable in magnitude to the 3D methods. The magnetic field had a limited effect on the isocentre measurements, the largest difference observed in the spoke shots between 1.5 and 0 T (approximately 0.1 mm). It is worth noting, the removal of the background noise, from profiles where distortion interfered with ballbearing edges, produced FWHM size comparable to images which did not suffer from distortion.

Anterior coil attenuation for various gantry angles is shown in Table 2. Attenuation was measured as approximately 0.6% from G0 and ranged between 0.4 and 0.7% for listed gantry angles. For the TPS, the G0 attenuation was 0.8% and ranged between 0.7 and 1.0% across the gantry angles investigated. Measured and calculated attenuation were consistent to within 0.5%, with the largest discrepancy occurring at G30. Whilst the attenuation of the beam due to the anterior coil is small, the effect this has on out-of-field doses is not [32]. During patient treatments on the Unity system, where multiple gantry angles would be used, the effect of the anterior coil attenuation on the delivered dose would be negligible; however, the potential ESE should be investigated, and appropriate patient shielding should be provided [32]. Note that angular dependent output through the other attenuating objects (couch and cryostat) for this system have been published elsewhere [33].

X-ray beam quality measurements showed that at commissioning the beam energy, according to the TPR_20,10_, matched the TPS within 0.5%. The measured value (0.705 ± 0.001) was slightly higher than those reported by Snyder et al*.* (0.704) [4], Woodings et al*.* (0.701) [6] and van Asselen et al*.* (0.701 ± 0.002) [16]; however, was within 0.6%. Interestingly, the dose map comparison at this beam quality to the TPS, Fig. 5a, highlights the opposite where the measured profile appeared slightly less peaked than the calculated. The differences presented in Fig. 5a were consistent for several deliveries, suggesting that measurement uncertainty was not the cause. However, given the subtlety of the difference, with gamma results above 95.0%, and the difficulties associated with attempting to improve results, no changes to the beam were made. Although, with the introduction of a new magnetron, the TPR_20,10_ decreased approximately 0.4% (0.703 ± 0.001) and better agreement of the beam shape was achieved against the TPS, Fig. 5b, and the TPR_20,10_ against other reported values [4, 6, 16].

Note for consistency with the TRS398 protocol [15], a 10.0 × 10.0 cm^2^ field at an SCD of 147.5 cm required a field of 9.7 × 9.7 cm^2^ at isocentre; however when a 10.0 × 10.0 cm^2^ field at isocentre was set, the change in measured TPR_20,10_ was negligible. TPR_20,10_ was insensitive to the change in magnetic field strength, consistent with the work of previous investigators [6, 16, 17]. For the same magnetron the TPR_20,10_ from G0 and G90 were equal within the measured standard deviation of the G0 measurements. The beam hardening effect of the tank wall was assumed to be negligible compared to that of the aluminium cryostat for these measurements; however, would still introduce uncertainty in the measurement.

Reference dosimetry as performed from G90 using the TPR_10,5_ adjustment was found to be reproducible, with a coefficient of variation (COV) of 0.3%, and accurate against the nominal value of 1.000 Gy per 100 MU. This method can therefore be argued to be suitable for determination of reference output. The use of the TPR_10,5_ reading from G0 to correct G90 readings assumes comparable beam quality between the two angles, which may not be the case due to variations in cryostat and fill. However, it can be assumed from the consistency of TPR_20,10_ from G0 and G90 that a change in TPR_10,5_ would also be negligible. Output measurements from G0 showed larger differences from the nominal value of 1.005 Gy per 100 MU and were more varied (COV = 0.5%) compared to the G90 technique; however, were still deemed adequate as a routine check.

Measured correction factors for the two identical Farmer type chambers, at depths 5.0 cm and 10.0 cm, were consistent with values reported by other investigators for the same chamber type (0.997 ± 0.002) [16]. After applying the dose conversion factor ${c}_{\tilde{B }}$ of 0.995 [16], the combined correction factors (${k}_{{B}_{\parallel },Q}$) were consistent with the work of O’Brien et al*.* (0.994 ± 0.001) [17] and van Asselen et al*.* (0.992 ± 0.002) [16]. The dose conversion factor is constant at depths ranging from 5.0 to 25.0 cm [17], and independent of the SSD differences discussed in this work.

Although the ${k}_{{B}_{\parallel },Q}$ correction factors for the two depths differ by 0.5%, they agree with published values at 10.0 cm within the measurement uncertainty of van Asselen et al*.* [16]. This suggests the magnetic field correction factor ${k}_{{B}_{\parallel },Q}$ is independent of depth and argues for the potential use of published values at either calibration depth. This is not surprising given the previous work of O’Brien et al*.* [34], where the dose-response of ionization chambers in a magnetic field environment tended to be depth dependent for small fields only. The lack of a reference chamber used in the determination of factors here will increase uncertainty, as consistent machine output between 0 and 1.5 T measurements cannot be guaranteed. However, this should not interfere with an assessment of depth dependence of the factors, as readings at the depths were acquired consecutively and output fluctuations were minimal on a given day.

Strictly speaking for Unity users calibrating machines in a similar manner to this work, magnetic field correction factors at 10.0 cm depth should be used, due to the measurement setup requirements. Ideally, reference dosimetry should be performed directly at isocentre from G90 and should this be achievable at 5.0 cm depth, with future vendor-designed 1D tanks, the corresponding factors should be correctly determined [21] and applied. The consistency of TPR_20,10_ between G0 and G90 supports measuring these factors from G0, in agreement with previous Monte Carlo simulations [18].

Initial commissioning of the Elekta supplied TG119 IMRT plans failed the departmental criteria of 2.0%, 2.0 mm (Tables 3, 4, 5) with average pass rates of 91.7 ± 7.0, 88.2 ± 11.1 and 87.1 ± 9.4% for the Octavius perpendicular (composite comparison), film perpendicular and film composite measurements, respectively. Of note were the poor results for the Multi-Target plan and the second prostate plan for all three QA techniques. At 3.0%, 3.0 mm gamma (as recommended during physics validation) results were initially considered acceptable with average rates above 95.0% for the three methods, consistent with previous investigators reporting with the same devices and criteria [2, 35]. Similar gamma results with criteria of 3.0%, 2.0 mm, as per AAPM TG-218 [36], were observed. The in-house stereotactic plans failed the departmental tolerance of 2.0%, 2.0 mm and even highlighted beam delivery issues at 3.0%, 3.0 mm. During segment-by-segment delivery of the stereotactic plans to the MVI panel, Fig. 6, an issue in the MOSAIQ sequencer was discovered, wherein it was applying an additional guard leaf rule to what was already set by the TPS. This occurred in part due to variation in leaf thickness at isocentre of the Unity MLCs (approximately 7.0 mm) from that for conventional systems (5.0 mm). Once rectified, all plans passed above 95.0% at 2.0%, 2.0 mm gamma criteria, with average passes of 98.3 ± 1.8, 97.6 ± 1.6 and 97.2 ± 1.3% for the Octavius perpendicular, film perpendicular and film composite deliveries, respectively. These values compare well to other investigators using the 1500^MR^ array [35] and others using the ArcCheck®-MR device [4, 7]; however, for those works a less strict criteria was reported. For array beam-by-beam analysis, the beams for all plans had pass rates greater than 94.5% at 2.0% (local dose), 2.0 mm gamma criteria.

These results show that the PSQA techniques above can be used to determine issues with plan delivery. Stereotactic-type plans were clearly more sensitive to the guard leaf error, due to the small segment sizes in these plans. These small-field plans would also be expected to be more sensitive to other beam shaping issues. As such it would be beneficial for new sites to perform their own measurements of such plans, as well as the Elekta TG119 plans, during commissioning. Segment-by-segment delivery of these stereotactic plans, to the MVI, would also be useful to help discern issues with field shapes, as shown above. Note that an alternate method for detecting such issues would be through analysis of the auto-generated treatment record files (TRFs) [37, 38]. Comparison of set leaf positions in the TPS could be compared against delivered position in TRFs, to highlight the errors discovered in this work.

For the PSQA procedures, dose is calculated in the planning system to a homogeneous water phantom. Previous investigators alluded that this may be inadequate to highlight modelling issues [39]. Additionally, the audit process by the Australian Clinical Dosimetry Service (ACDS) requires unforced densities for their phantom when performing similar measurements. For the PSQA procedures above, the use of unforced densities in the TPS could potentially add uncertainty, due to day-to-day variations in setup which would not be present in the reference scan. Furthermore, forcing the RED of the solid water contour to 1.000 is more convenient for routine practice, is accounted for during the film calibration and was determined to be acceptable through comparison of a measured water PDD to that of the solid water under the same conditions. Finally, comparisons between film and TPS dose, with and without water sprayed on the film, revealed no statistically significant differences in gamma results between the two when following the QA methodology above.

End-to-end results for both ATP and ATS created plans had pass rates > 97.0% at 2.0% local dose and 2.0 mm DTA. Snyder et al*.* [4], using a commercial thorax E2E phantom with film, reported a pass rate of 98.0% for an ATS plan at 7.0% dose difference and 4.0 mm DTA. Due to the resolution of the 3D printer, the tumour surrogates housing the film were not airtight. This may have been of benefit as it meant that water was able to surround the film, thereby reducing uncertainties due to airgaps generating increased ERE dose. Although not a comprehensive set, the results above indicate that the use of in-house developed/3D-printed phantoms can be of benefit on the Elekta Unity MRL for End-to-End purposes.

## Conclusion

In this work we performed commissioning measurements on an Elekta Unity MRL and aimed to provide guidance for others when implementing this machine, without reliance on vendor supplied equipment or results. A subset of our commissioning work was presented, with the use of commercial equipment, simple in-house phantoms and adaptations on standard methodologies having been discussed. We determined the MV alignment phantom can be used for gantry angle confirmation at angles other than 270.0° and visual confirmation of the set angle can be achieved within tolerance using MVI images of the phantom. This phantom, and in-house methods, can be used to determine an isocentre size comparable to vendor recommended tech niques. Additionally, the attenuation due to the anterior imaging coil was found to be negligible and measured values agree with TPS calculations. When vendors are designing commercial 1D water tanks, the requirements of different clinics should be considered, such as linac calibration depths and beam quality measurements. Gafchromic film and the Octavius 1500^MR^ array can be used to highlight issues with plan deliverability, when appropriate gamma criteria are set, and commissioning of small field plans should be considered by clinics to help highlight differences between measurements and TPS calculations. Finally, E2E testing and IMRT deliveries met internal criteria prior to clinical release of the machine. We hope this work aids other centres in the commissioning of MR Linacs.

## Data Availability

All data relevant to this article can be made available upon request.
